# Efficient induction of CD25^- ^iTreg by co-immunization requires strongly antigenic epitopes for T cells

**DOI:** 10.1186/1471-2172-12-27

**Published:** 2011-05-05

**Authors:** Shuang Geng, Yang Yu, Youmin Kang, George Pavlakis, Huali Jin, Jinyao Li, Yanxin Hu, Weibin Hu, Shuang Wang, Bin Wang

**Affiliations:** 1 State Key Laboratory for Agro-Biotechnology China Agricultural University, Beijing 100193, China; 2 Center for Cancer Research, National Cancer Institute at Frederick, Frederick, Maryland 21702, USA; 3 College of Veterinary Medicine, China Agricultural University, Beijing 100193, China; 4 Department of Microbiology and Immunology, College of Medicine, The University of Illinois at Chicago, Chicago, Illinois, USA; 5 National Cancer Institute at Frederick, Frederick, Maryland, USA

## Abstract

**Background:**

We previously showed that co-immunization with a protein antigen and a DNA vaccine coding for the same antigen induces CD40^low ^IL-10^high ^tolerogenic DCs, which in turn stimulates the expansion of antigen-specific CD4^+^CD25^-^Foxp3^+ ^regulatory T cells (CD25^- ^iTreg). However, it was unclear how to choose the antigen sequence to maximize tolerogenic antigen presentation and, consequently, CD25^- ^iTreg induction.

**Results:**

In the present study, we demonstrated the requirement of highly antigenic epitopes for CD25^- ^iTreg induction. Firstly, we showed that the induction of CD25^- ^iTreg by tolerogenic DC can be blocked by anti-MHC-II antibody. Next, both the number and the suppressive activity of CD25^- ^iTreg correlated positively with the overt antigenicity of an epitope to activate T cells. Finally, in a mouse model of dermatitis, highly antigenic epitopes derived from a flea allergen not only induced more CD25^- ^iTreg, but also more effectively prevented allergenic reaction to the allergen than did weakly antigenic epitopes.

**Conclusions:**

Our data thus indicate that efficient induction of CD25^- ^iTreg requires highly antigenic peptide epitopes. This finding suggests that highly antigenic epitopes should be used for efficient induction of CD25^- ^iTreg for clinical applications such as flea allergic dermatitis.

## Background

The inducible regulatory T cells, or iTreg, differ from the naturally regulatory T cells (nTreg) in that the former are generated in the periphery through encounter with environmental antigens. It is also believed that iTreg play non-overlapping roles, relative to nTreg, in regulating peripheral tolerance [1-3]. Most iTreg reported to date have been CD25^+ ^cells (CD4^+^CD25^+^Foxp3^+^), and it is well established that their induction requires suboptimal stimulation of the T cell receptor (TCR) and cytokines TGF-β and IL-2 [3]. The CD25^+ ^iTreg thus appear to derive primarily from weakly stimulated CD4^+ ^T cells.

We previously identified a different subset of iTreg in mice that is CD25^- ^(CD4^+^CD25^-^Foxp3^+ ^and IL-10^+^TGF-beta^+^IFN-γ^-^). The CD25^- ^iTreg were induced after co-immunization using a protein antigen and a DNA vaccine encoding the same antigen [4-7]. Unlike that of the CD25^+ ^iTreg, the induction of the CD25- iTreg involved the generation of CD40^low ^IL-10^high ^tolerogenic dendritic cells (DCs), which in turn stimulated CD25^- ^iTreg in an antigen-specific manner [4]. We further showed in mouse models that this subset of iTreg was potentially useful as a therapeutic for allergic and autoimmune diseases, such as asthma, flea allergic dermatitis (FAD), and type 1 diabetes (T1D) [5-7].

While the requirement for weak antigen stimulation is well established for the induction of CD25^+ ^iTreg, it is unclear whether the same is true for the induction of CD25^- ^iTreg. Addressing this question will allow us not only to further differentiate the two subsets of iTreg, but also to maximize the tolerogenicity of co-immunization by choosing T cell epitopes of appropriate antigenicity. In this report, we show that strong antigen stimulation is required for efficient induction of CD25^- ^iTreg.

## Results

### MHC-Ag:TCR interaction is required for induction of CD25^- ^iTreg

**Figure 1:**
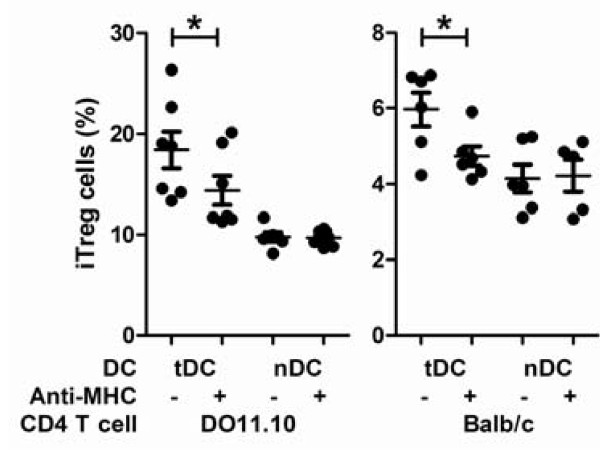
**MHC II blocking reduces CD25^- ^iTreg induction**. Purified CD4^+ ^T cells from Balb/c DO11.10 mice or OVA_323-339_-sensitized Balb/c mice were cultured with purified tolerogenic DCs (tDC) from co-immunized Balb/c mice or naïve DCs (nDC) from naïve Balb/c mice, in the presence or absence of anti-MHC-II blocking mAb. CD25^- ^iTreg cells (CD4^+^CD25^-^Foxp3^+^) were counted on day 7 as percentage of CD4^+^CD25^- ^T cells *, *p *< 0.05 by the Mann-Whitney U test. Shown is one of three independent experiments with similar results. Each dot represents one mouse, n ≥ 5.

To test whether the MHC-Ag:TCR interaction is required for the induction of CD25^- ^iTreg, we employed an *in vitro *iTreg induction system. It involved culture of CD4^+ ^T cells together with co-immunization-induced tolerogenic DCs that presented the dominant epitope of hen ovalbumin, OVA_323-339_. Using either clonotypic CD4^+ ^T cells from DO11.10 Balb/c mice or polyclonal CD4^+ ^T cells from ovalbumin-sensitized Balb/c mice, we found that the induction of CD25^- ^iTreg in either case could be blocked by anti-MHC-II antibody and, therefore, was MHC-II-dependent. Thus, antigenic stimulation is essential for the induction of CD25^- ^iTreg (Figure 1).

### Highly antigenic epitopes are required for efficient induction of highly active CD25^- ^iTreg

**Figure 2:**
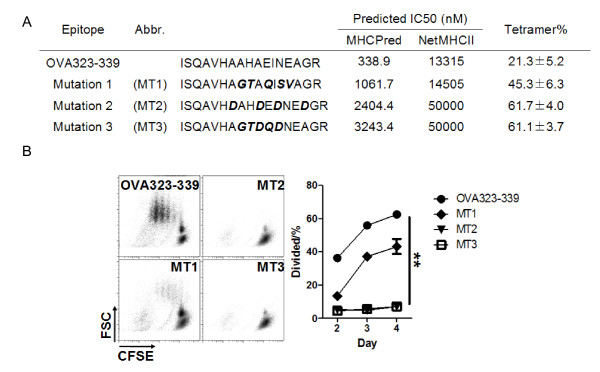
**OVA_323-339 _mutations reduce antigenicity for T cells**. *A*, Summary of OVA_323-339 _mutations, their predicted MHC II binding affinities, and experimental result from tetramer competition assays. Percent of tetramer binding was calculated as: number of tetramer-positive T cells in the presence of a competing peptide epitope / number of tetramer-positive T cells in the absence of a competing peptide epitope ×100%. *B*, Proliferation of CFSE-labeled DO11.10 CD4^+ ^T cells co-cultured for 4 days with tolerogenic DCs presenting an indicated epitope. The line plots summarize the results from three independent experiments. **, *p *< 0.01, n = 3.

To further determine how antigenicity affects CD25^- ^iTreg induction, we generated a set of mutated epitopes from OVA_323-339_. Using a tetramer staining-based epitope competition assay, we assessed the affinity of each of the mutated epitopes for MHC II. The result showed the order of affinity to be OVA_323-339 _> MT1 > MT2 = MT3 (Figure 2A). Consistently with this result, *in vitro *T cell proliferation assays using DO11.10 CD4^+ ^T cells showed a similar order in T cell stimulating activity (Figure 2B). We therefore selected the epitopes OVA_323-339_, MT1, and MT2 as probes for antigenicity studies.

**Figure 3:**
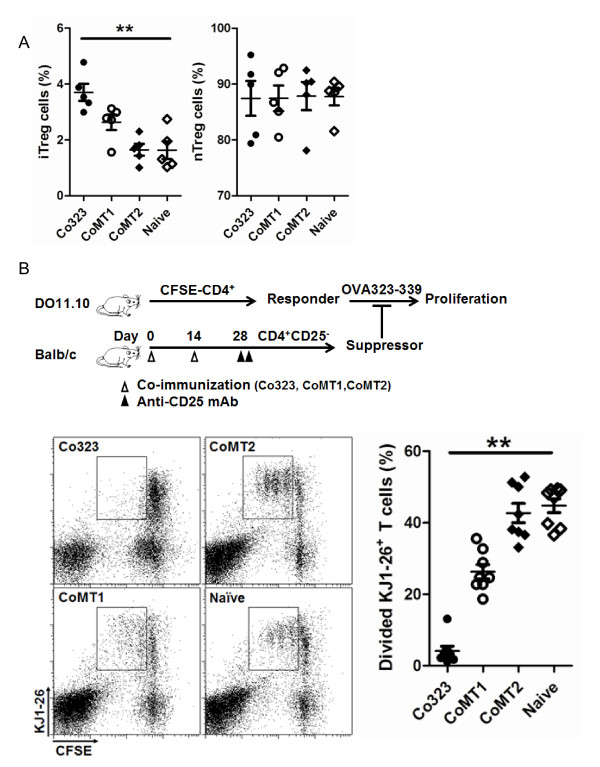
**Induction of CD25^- ^iTreg cells by co-immunization depends on epitope affinity**. *A*, CD25^- ^iTreg (CD4^+^CD25^-^Foxp3^+^) and nTreg (CD4^+^CD25^+^Foxp3^+^) induced in Balb/c mice following co-immunization were counted by flow cytometry and calculated as percentage of Foxp3^+ ^cells in CD4^+^CD25^- ^and CD4^+^CD25^+ ^T cells, respectively. Naïve, non-immunized mice. **, *p *< 0.01, n ≥ 5. Difference was calculated among all groups. Each point represents one mouse. Shown is one of three independent experiments with similar results. *B*, Induction of highly suppressive CD25^- ^iTreg cells by co-immunization depends on epitope antigenecity. CFSE labeled DO11.10 CD4^+ ^T cells were co-cultured with co-immunization-induced CD25^- ^iTreg, in the presence of OVA_323-339_. Proliferation was determined by flow cytometry as divided KJ1-26^+ ^cells versus total KJ1-26^+ ^cells. **, *p *< 0.01, n ≥ 5. Each point represents one mouse. Difference was calculated among all groups. Shown is one of three independent experiments with similar results.

To that end, Balb/c mice (I-Ad^+^) were treated by co-immunization using the DNA and protein combination corresponding to the OVA_323-339_, MT1, or MT2 epitope (designated as Co323, CoMT1, or CoMT2). Seven days after the treatment, splenocytes were isolated and analyzed for CD25^- ^iTreg induction. The result showed increased frequency of Foxp3^+ ^cells in the CD4^+^CD25^- ^(CD25^- ^iTreg), but not the CD4^+^CD25^+ ^(nTreg), cell population in the treated mice, compared to that of untreated control mice (naïve) (Figure 3A). Importantly, the magnitude of increase followed the order of Co323 > CoMT1 > CoMT2, suggesting that efficient induction of CD25^- ^iTreg by co-immunization required highly antigenic epitopes.

To further determine the impact of antigenicity on the function of CD25^- ^iTreg, we compared the suppressive activity of CD25^- ^iTreg induced by Co323, CoMT1, and CoMT2 using an *in vitro *suppression assay. All CD25^- ^iTreg cells suppressed the OVA_323-339_-specific proliferation of reporter CD4^+ ^T cells in co-culture as expected. However, their relative suppressive activity followed the same order of Co323 > CoMT1 > CoMT2 (Figure 3B), suggesting that more antigenic epitopes also induce functionally more active CD25^- ^iTreg cells.

**Figure 4:**
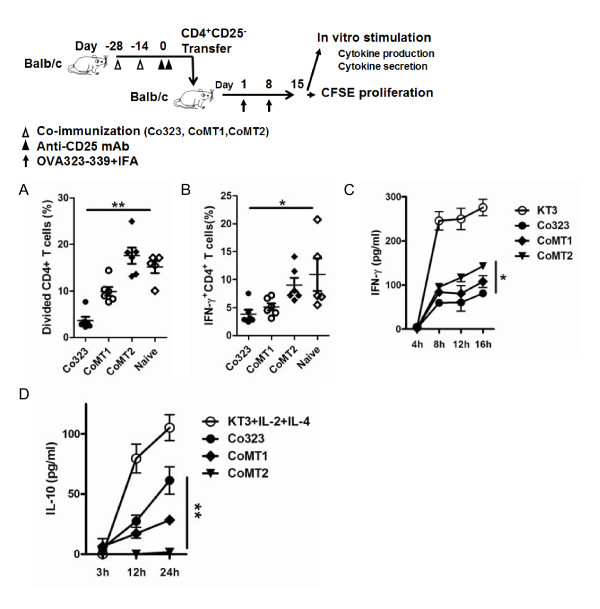
**Adoptive transfer of CD25^- ^iTreg cells suppresses T cell response in recipient mice**. CD4^+^CD25^- ^T cells from OVA_323-339_, MT1, or MT2 co-immunized, or from naïve Balb/c, were adoptively transferred to naïve Balb/c. The activity of the donor CD25^- ^iTreg was assessed by sensitizing the recipients with OVA_323-339 _in IFA. *A*, CD4^+ ^T cells were isolated from the recipient after sensitization. The cells were labeled with CFSE and restimulated with OVA_323-339 _in culture. Divided cells were identified by CFSE dilution and counted by flow cytometry. The result is expressed as a percent of total CFSE^+ ^T cells. Shown is one of three independent experiments of similar results. *B*, CD4^+ ^T cells were isolated from the recipients after sensitization and intracellularly immunostained for IFN-γ. IFN-γ^+^CD4^+ ^T cells were counted by flow cytometry and calculated as a percent of total CD4^+ ^T cells. Shown is one of three independent experiments of similar results. *C *&*D*, IFN-γ and IL-10 secretion in the supernatant of restimulated T cells. Anti-CD3 mAb (KT3) or KT3 + IL-2 + IL-4 was used in positive controls for induction of indicated cytokines. Shown is one of three independent experiments of similar results. *, *p *< 0.05, **, *p *< 0.01, n ≥ 5. Difference was calculated among all groups (A, B) or among Co323, CoMT1, and CoMT2 (C, D).

To repeat this observation *in vivo*, we adoptively transferred CD25^- ^iTreg induced with the different epitopes into Balb/c mice and then attempted to sensitize the animals with OVA_323-339 _in incomplete Freund's adjuvant (IFA). One week later, we isolated splenic CD4^+ ^T cells from the sensitized mice and measured recall activation of CD4^+ ^T effector cells by an *in vitro *restimulation assay. Again, although all transferred CD25^- ^iTreg blocked the recall proliferation of T cells to some degree, their relative effectiveness varied with the inducing epitopes, in the order of Co323 > CoMT1 > CoMT2 (Figure 4A). Moreover, splenic CD4^+ ^T cells isolated from the recipients showed decreased expression of IFN-γ and increased expression IL-10, the extent of which also followed the same order (Figure 4, B-D). Taken together, these results show that highly antigenic epitopes are required for more efficient induction of highly suppressive CD25^- ^iTreg.

### Highly antigenic epitopes are also required for more effective prevention of flea allergic dermatitis

**Figure 5:**
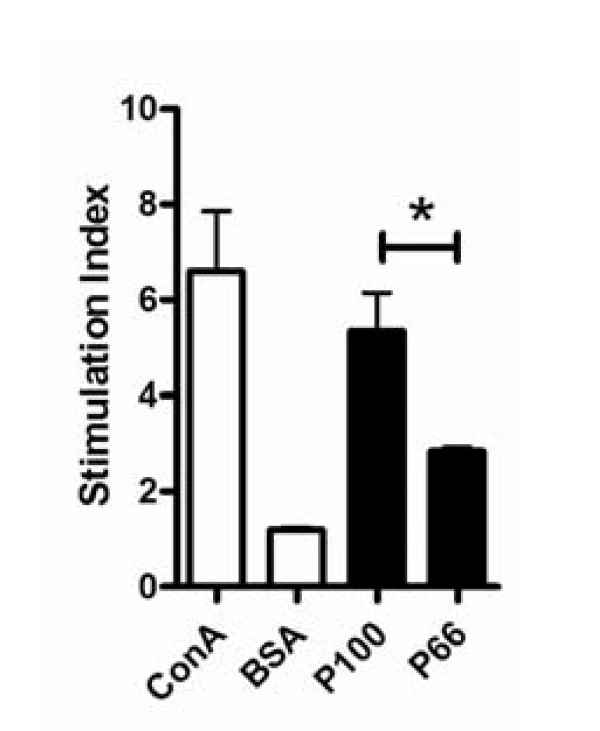
**P100 stimulates T cells more strongly than P66**. Splenic CD4^+ ^T cells from flea antigen immunized C57BL/6 mice were restimulated with P100 or P66 (5 μg/ml) in culture. T cell proliferation was determined by an MTT assay. Concanavalin A (1 μg/ml) and BSA (1 μg/ml) were used as positive and negative controls, respectively. *, *p *< 0.05, n = 3. Shown is one of three independent experiments with similar results.

Flea allergic dermatitis is a CD4^+ ^T cell-mediated allergic reaction to flea allergen [8,9]. To extend our finding to this disease model, we chose two antigenic epitopes from the flea allergen FSA1, namely P66 (amino acids 66-80) and P100 (amino acids 100-114). P100 was predicted to have a higher affinity to MHC II (I-Ab) than P66. We confirmed this prediction by sensitizing C57BL/6 mice (I-Ab^+^) with full-length FSA1, followed by an *in vitro *restimulation assay using one of the epitopes. We found that P100 indeed induced significantly more vigorous T cell proliferation than did P66 (Figure 5).

**Figure 6:**
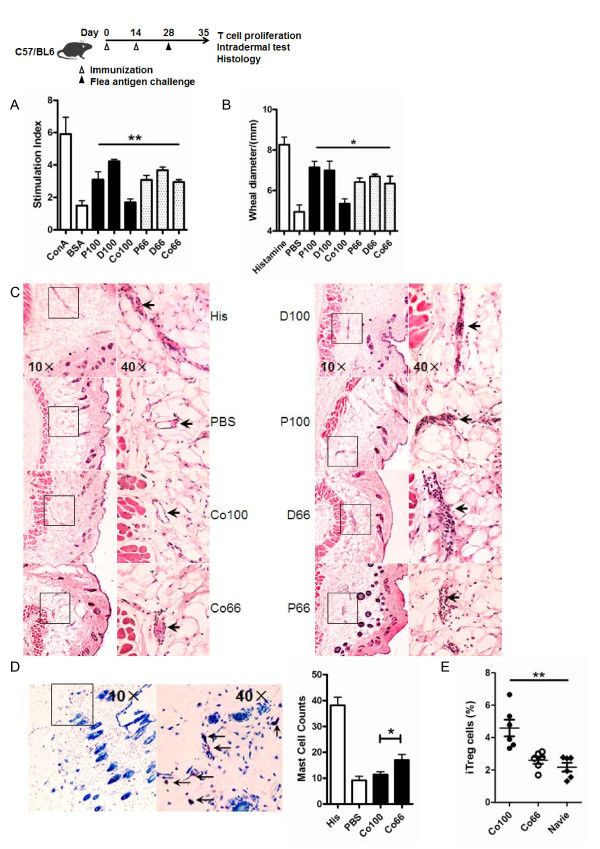
**Attenuation of skin reaction by co-immunization-induced CD25^- ^iTreg**. *A*, Flea antigen stimulated T cell proliferation. *B, In vivo *T cell response induced by flea-specific i.d. test. *C*, H&E staining of skin section. The black arrows indicate infiltrating T cells. *D*, Mast cell number and degranulation (black arrow) by Toluidine Blue staining. *E*, Seven days after co-immunizaiton, CD25^- ^iTreg cells were counted as a percentage of CD4^+^CD25^- ^T cells. Shown is one of three independent experiments with similar results. *, *p *< 0.05; **, *p *< 0.01, n ≥ 5. Difference was calculated among all immunized groups (A, B) or all groups (E).

To see whether the difference in antigenicity influences the induction of CD25^- ^iTreg cells by these two epitopes, we prophylactically treated C57BL/6 mice with co-immunization using the combination of DNA and protein vaccines targeting each epitope (designated as Co100 or Co66). Seven days after co-immunization, the animals were sensitized with flea saliva extracts, followed by a delayed-type hypersensitivity assay to determine to which extent the prophylactic co-immunization had prevented the development of an allergic reaction. Both the wheal size analysis and histological examination showed a stronger protective effect by Co100 than by Co66, as indicated by smaller wheal diameters (Figure 6B) and fewer mononuclear infiltrates (Figure 6C) at the reaction site. The Co100-treated mice also had fewer mast cells and a lower level of degranulation at the reaction site (Figure 6D). *In vitro *recall activation also confirmed a weaker T cell response in the Co100 group (6*A*). Importantly, P100 also induced more CD25^- ^iTreg than P66 (Figure 6E), suggesting that P100 protected animals more effectively by inducing more CD25^- ^iTreg.

**Figure 7:**
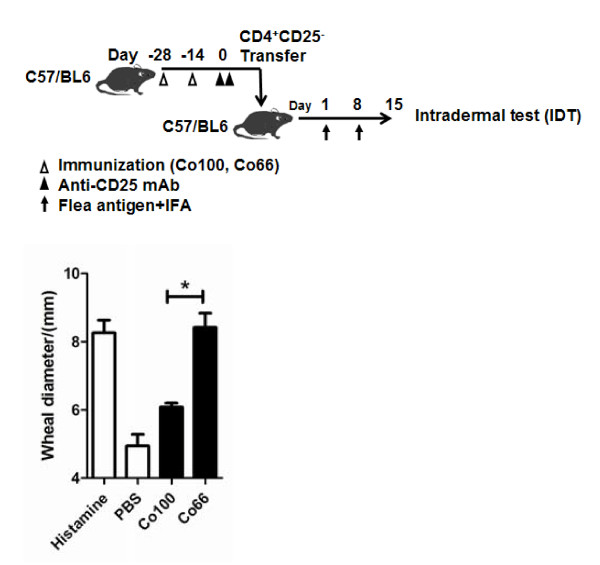
**Adoptive transfer of CD25^- ^iTreg suppresses skin response *in vivo***. CD25^- ^iTreg from Co100 or Co66 immunized mice were adoptively transferred into FSA1-sensitized mice. The recipients were then challenged with flea antigens (skin test). Histamine and PBS were used as positive and negative controls for the skin test, respectively. *, *p *< 0.05, n = 3. Shown is one of three independent experiments with similar results.

To determine whether this is indeed the case, we adoptively transferred CD25^- ^iTreg cells induced by Co100 or Co66 into FSA1-sensitized mice and challenged the recipients with flea antigens. Again, recipients receiving Co100-induced CD25^- ^iTreg cells showed significantly reduced DTH response than those receiving Co66-induced counterpart (Figure 7). Collectively, these results confirm in this disease model that highly antigenic epitopes are required for more efficient induction of therapeutic CD25^- ^iTreg.

## Discussion

We have shown for the first time that efficient induction of highly active CD25^- ^iTreg cells requires highly antigenic epitopes for T cells. The finding is based on 1) anti-MHC-II mAb blocked the induction CD25^- ^iTreg cells *in vitro *(Figure 1); 2) OVA_323-339 _mutants with decreased antigenicity for T cells showed decreased ability to induce active CD25^- ^iTreg cells (Figures 2,3,4); and 3) a similar observation was made in a mouse model of flea allergic dermatitis, where CD25^- ^iTreg cells induced by a more antigenic epitope were also more effective in preventing the development of the disease (Figures 5,6,7).

Given that flea allergic dermatitis is a disease driven primarily by effector CD4^+ ^T cells, our observation that the CD25^- ^iTreg cells are effective at suppressing this disease suggests that these cells function, at least partly, by suppressing effector CD4^+ ^T cells. A similar mechanism of action has long been described for other types of Treg cells, including both the natural (thymus-derived) and induced CD4^+ ^Treg cells that display the CD25^+^Foxp3^+ ^phenotype [10-15]. Thus, despite displaying a different phenotype (CD25^-^) and requiring strong antigenic stimulation for induction, the CD25^- ^iTreg cells described in the present work may exert their regulatory function similarly to the other types of Treg cells, i.e. by inhibiting the activity of other immune cells, particularly T cells and antigen presenting cells, in a cell contact-dependent and/or -independent manner. Future investigation is needed to determine whether this is indeed the case.

iTreg cells are potentially useful as therapeutics for allergy, autoimmune diseases, and transplant rejection [7,16-18]. The present study thus has the translational importance by uncovering the need for choosing highly antigenic epitopes for effective induction of CD25^- ^iTreg. At present, immunosuppressant treatment is the only means to control immune disorders and pathology, which is unfortunately associated with many side effects, including increased risk of infection and cancer [19,20]. *In vivo *induction of CD25^- ^iTreg cells, which are antigen-specific, provides a novel means of controlling immune diseases while avoiding global immunosuppression.

Despite their therapeutic potential, it remains to be determined whether the CD25^- ^iTreg cells are functionally stable for use in therapy. It is worth noting that CD25^+ ^iTreg, which are induced by TGF-beta [21,22]; have been shown to lose [23] or maintain [24] the Foxp3^+ ^phenotype, and suppressive activity, depending on certain conditions. In our system, the CD25- iTreg cells appeared to remain Foxp3^+ ^and function well 14 days after being induced in vivo via co-immunization, as judged by both the *in vitro *(Figure 3B and 6A) and *in vivo *(Figure 4 and 7) assays. Nonetheless, these cells need to be followed for a much longer-term in order to assess their long-term functional stability.

It is interesting to note that strong antigenic epitopes are a known requirement for efficient induction of effector T cells. How does this same requirement differentiate iTreg versus conventional T cells? Our published work suggests that the differentiating factor is likely to be the tolerogenic DCs generated under the condition of co-immunization, which serve as an endogenous tolerogenic adjuvant [4-7]. It is therefore likely that either the strongly antigenic epitopes are involved in the generation of tolerogenic DCs or they preferentially stimulate iTreg in combination with the tolerogenic DCs. The two possibilities are not mutually exclusive. We are in process to investigate these possibilities.

The complex of MHC-epitope (pMHC):TCR is pivotal in T cell immunity, and a strong interaction between pMHC and TCR induces stronger T cell activation [25,26]. But controversy remains, about within this complex, which affinity is effective, epitope to MHC, or pMHC to TCR. However the issue is of theoretical importance only in a system that employs clonotypic T cells, such as the DO11.10 TCR-transgenic mice; in contrast, in normal animals, the affinity of epitopes to MHC is presumably more important because the resulting pMHC spontaneously selects responding T cells with the highest-affinity TCR from a repertoire of T cells. Consistent with this assumption, our data showed that epitopes with predicted higher affinity to MHC also induced stronger T cell responses in both Balb/c mice and the mouse model of flea allergic dermatitis. Thus, in the limited wild-type animals that we have tested, it appears that strong antigenicity, and efficient induction of CD25^- ^iTreg, can be predicted from the affinity of epitope to MHC.

## Conclusions

In conclusion, we believe that highly therapeutically effective CD25^- ^iTreg may be induced by co-immunization targeting one or several disease-related or specific antigens, and by selecting antigenic epitopes of highest antigenicity for T cells as the immunogen.

## Methods

### Animal and reagents

Balb/c and C57/B6 mice were purchased from Beijing Vital Laboratory Animal Technology Company, Ltd. (Beijing, China) and Balb/c, DO11.10 were from SLAC Laboratory Animal (Shanghai, China) and maintained under pathogen-free conditions. All animal experiments were approved by the Committee of Experiment Animals of China Agricultural University. Peptides were synthesized by Scipeptide Ltd. (Shanghai, China). Antibodies for flow cytometry were purchased from BD Biosciences (CA, USA). Flea saliva extracts were purchased from China Medicines Corporation (Beijing, China).

### Epitope design

The dominant epitope of hen ovalbumin for I-Ad (OVA_323-339_: ISQAVHAAHAEINEAGR) was mutated as reported [27] and predicted with online servers MHCPred (http://www.ddg-pharmfac.net/mhcpred/MHCPred/) and NetMHCII (http://www.cbs.dtu.dk/services/NetMHCII/). Affinity scores were presented as IC50 (nM). The epitopes of flea salivary antigen 1 (FSA1, Swiss-Prot: Q94424.3) for I-Ab (P100: GPDWKVSKECKDPNN and P66: QEKEKCMKFCKKVCK) were selected using MHCPred. Corresponding DNA vaccines coding for OVA_323-339_, MT1, MT2, P100, and P66 were constructed with the pVAX1 vector, designated as pVAX1-OVA, pVAX1-MT1, pVAX1-MT2, D100, and D66.

### Antigen sensitization

Mice were immunized by subcutaneous injection (s.c.) twice on days 0 and 7 with 100 μg peptide emulsified in 100 μl IFA (Sigma-Aldrich Inc. San Louis, USA).

### Tolerogenic co-immunization

Balb/c mice were injected intramuscular (i.m.) on days 0 and 14 with 100 μg each of OVA_323-339 _and pVAX1-OVA, MT1 and pVAX1-MT1, or MT2 and pVAX1-MT2. C57BL/6 mice were similarly injected with P100 and D100, or P66 and D66.

### MHC-II blocking

Purified CD4^+ ^T cells (5 × 10^5^, R&D System, Minneapolis, USA, MAGM202) from Balb/c DO11.10 mice or OVA_323-339 _sensitized Balb/c mice were cultured with purified DCs (1 × 10^5^, Miltenyi Biotec, Gladbach, Germany, 130-052-001) from co-immunized (pVAX1-OVA plus OVA_323-339_) Balb/c mice. The cells were cultured for 7 days with or without anti-MHC II mAb (M5/114.15.2, eBioscience, San Diego, USA).

### Flow Cytometry

CD4^+^CD25^-^Foxp3^+ ^iTreg were detected by immunostaining with anti-CD4-FITC, anti-CD25-APC, and anti-Foxp3-PE mAbs. Intracellular IFN-γ was detected in monensin-blocked and anti-CD3 and anti-CD28 stimulated T cells by intracellular staining with anti- IFN-γ-PE mAb. Data were collected with a BD FACSCalibur and analyzed with Flowjo (Tree Star, Ashland, USA). The supernatant of cultured T cells was also analyzed for IFN-γ and IL-10 using the FlexSet Beads Assay (BD Biosciences).

### Tetramer competition assay

PE-conjugated OVA_323-339_-loaded I-Ad tetramer (NIH Tetramer Core Facility) was competed with OVA_323-339 _or a mutant peptide by incubation of 2 × 10^5 ^DO11.10 T cells, the OVA_323-339 _tetramer, and a competing peptide together for 5 minutes. Five volumes of medium with 10% FCS were added to stop the competition. Cells were washed 3 times and immediately analyzed for PE-positive T cells by flow cytometer. Data was presented as percent of tetramer-bound T cells in total T cells.

### T cell proliferation

MTT-based and CFSE-based T cell proliferation assays were performed as described before [7,27].

### *In vitro *suppression assay

OVA_323-339_-specific CD4^+ ^T cells from DO11.10 mice spleen were labeled with CFSE (responder cells) and co-cultured with co-immunization-induced CD4^+^CD25^- ^T cells at a 1:1 ratio (2 × 10^5 ^each). OVA_323-339_-specific proliferation of the responder cells was analyzed by CFSE dilution on day 4 using a FACScalibur. To block nTreg *in vivo*, two 10 μg dose of anti-CD25 mAb (clone 3c7, eBioscience) were injected intravenously (i.v.) into co-immunized mice at -48 h and -24 h before CD25^- ^iTreg isolation.

### *In vivo *suppression assay

Balb/c mice were injected (i.v.) with co-immunization-induced CD25^- ^iTreg (2 × 10^6^) on day 0. On days 1 and 8, the mice were repeatedly sensitized for the same antigen. On day15, the mice were sacrificed and splenic T cells were isolated and analyzed for recall activation by the T cell proliferation assays.

Intradermal test (IDT) and histology

Antigen-sensitized C57BL/6 mice were challenged intradermally (i.d.) with 10 μg of FSA (Greer Laboratories) on the nonlesional lateral thorax skin. PBS is used as a sham control and histamine is used as a positive control. The diameter of the skin reaction was measured within 30 min after challenge using a calibrated micrometer. Skin samples were collected within 30 min of antigen challenge, fixed in 4% paraformaldehyde, embedded in paraffin, and sectioned. Antigen retrieval was accomplished by boiling the slides in 0.01 M citrate buffer (pH 6.0), followed by staining with H&E for T cells or toluidine blue for mast cells.

### Statistical analysis

Pair-wise comparison was made using Student's t test (Figures 5, 6D, 7) or the Mann-Whitney U test (Figure 1). Comparison among three or more groups was made by ANOVA (Figures 2B, 3, 4, 6E). Difference is considered statistically significant if *p *< 0.05. * indicates *p *< 0.05; **, *p *< 0.01.

## Authors' contributions

SG and YY contributed equally to this work. GP provided tetramer and critical suggestions for this work. YH provided expert pathology criticism on histology work. YK, JL, HJ provided critical suggestions on experiment design. WH and SW participated in experiment execution.
